# Misuse of Artemisinin Combination Therapies by Clients of Medicine Retailers Suspected to Have Malaria Without Prior Parasitological Confirmation in Nigeria

**DOI:** 10.15171/ijhpm.2017.122

**Published:** 2017-11-01

**Authors:** Ernest Nwokolo, Chinazo Ujuju, Jennifer Anyanti, Chinwoke Isiguzo, Ifeanyi Udoye, Elamei BongosIkwue, Onoriode Ezire, Mopelola Raji, Wellington A. Oyibo

**Affiliations:** ^1^Society for Family Health, Abuja, Nigeria.; ^2^ANDI Centre of Excellence for Malaria Diagnosis, College of Medicine, University of Lagos, Lagos, Nigeria.

**Keywords:** ACT Misuse, Malaria Case Management in Africa, Test Before Treatment, Private Medicine Vendors, Presumptive Malaria Treatment

## Abstract

**Background:** Prompt and effective case detection and treatment are vital components of the malaria case management strategy as malaria-endemic countries implement the testing, treating and tracking policy. The implementation of this policy in public and formal private sectors continue to receive great attention while the informal private retail sector (mostly the patent and propriety medicine vendors [PPMVs]) where about 60% of patients with fever in Nigeria seek treatment is yet to be fully integrated. The PPMVs sell artemisinin combination therapies (ACTs) without prior testing and are highly patronized. Without prior testing, malaria is likely to be over-treated. The need to expand access to diagnosis in the huge informal private health sector among PPMVs is currently being explored to ensure that clients that patronize retail drug stores are tested before sales of ACTs.

**Methods:** A cross-sectional multistage study was conducted among 1279 adult clients, 20 years and above, who purchased malaria medicines from 119 selected PPMVs in five administrative areas (States) of Nigeria, namely: Adamawa, Cross River, Enugu, Lagos and Kaduna, as well as the Federal Capital Territory, Abuja. Exit interviews using a standard case report questionnaire was conducted after the purchase of the antimalarial medicine and thick/thin blood smears from the clients’ finger-prick were prepared to confirm malaria by expert microscopy.

**Results:** Of the 1279 clients who purchased malaria medicines from the PPMV outlets, 107 (8.4%) were confirmed to have malaria parasites. The malaria prevalence in the various study areas ranged from 3.5% to 16%. A high proportion of clients in the various study sites who had no need for malaria medicines (84%-96.5%) purchased and used antimalarial medicines from the PPMVs. This indicated a high level of over-treatment and misuse of antimalarials. Common symptoms that are widely used as indicators for malaria such as, fever, headache, and tiredness were not significantly associated with malaria. Nausea/vomiting, poor appetite, chills, bitter taste in the mouth and dark urine were symptoms that were significantly associated with malaria among the adult clients (P<.05) but not fever (P=.06).

**Conclusion:** Misuse of ACTs following overtreatment of malaria based on clinical diagnosis occurs when suspected cases of malaria are not prior confirmed with a test. Non-testing before sales of malaria medicines by PPMVs will perpetuate ACT misuse with the patients not benefiting due to poor treatment outcomes, waste of medicines and financial loss from out-of-pocket payment for unneeded medicines.

## Background

Malaria in Nigeria is estimated to be responsible for 60% of outpatient visits to health facilities, 30% of childhood deaths, 25% of death in children less than one year of age and 11% of maternal deaths.^1^ The 60% report of malaria in outpatient visitation was largely due to presumptive clinical diagnosis. However, with current massive deployment of malaria control measures such as the widespread use of long lasting insecticide treated nets (LLINs), use of sulphadoxine pyremithamine as intermittent preventive treatment of malaria in pregnancy (IPTp), effective malaria case management with artemisinin combination therapies (ACTs), indoor residual spraying among others, the epidemiology of malaria in Nigeria appears to be changing with concomitant reduction in malaria rates among children presenting in health facilities.^2^

In 2009, the World Health Organization (WHO) recommended parasitological confirmation for all suspected cases of malaria before treatment.^3^ This recommendation was immediately implemented by the National Malaria Elimination Programme (NMEP) of the Federal Ministry of Health of Nigeria with the subsequent activation of the Malaria Diagnosis and Treatment Policy^1^ and the Malaria Diagnosis and Treatment Guideline.^4^ This policy is more operational in public health sector facilities and some formal private health facilities. The goal of effective case management is firstly to reduce morbidity and mortality by ensuring rapid, complete cure of *Plasmodium* infection, thus preventing the progression of uncomplicated malaria to severe and potentially fatal disease, as well as preventing chronic infection that leads to malaria-related anaemia; secondly, to curtail the transmission of malaria by reducing the human parasite reservoir; and thirdly to prevent the emergence and spread of resistance to antimalarial medicines. Artemisinin resistance has been reported in four countries in the South East Asia region, and the sustained potency of ACTs is a concern to the global community.^3^

In the private health sector, parasite-based diagnosis of malaria is infrequently done especially in the informal private retail sector that is dominated by the patent and propriety medicine vendors (PPMVs). Consequently, malaria medicines are sold and used without parasitological confirmation. This misuse of the ACTs could trigger resistance of the malaria parasites to these medicines, negate the need for investigating the actual cause of fever and importantly gloss over life-threatening diseases that could become fatal. Overall, the goal of providing effective case management of malaria, expanding access to parasitological testing and the attainment of national and global targets could be threatened. Evidence of malaria overdiagnosis and over-treatment has been reported in Nigeria,^2^ Tanzania,^5,6^ Afghanistan,^7^ and Ghana.^8^

ACTs are sold over-the-counter in registered Pharmacies and by PPMVs in Nigeria and it is easy for persons with no malaria confirmatory diagnosis in public health facilities to purchase these medicines if not convinced about the outcome of the test. In addition, about 60% of persons with fever in Nigeria are first seen by the PPMVs.^9^ Thus, the private retail sector is an important platform to provide parasitological confirmation of malaria. The private retail sector that sells medicines is in different categories namely: (*a*) Community Pharmacy that is managed by Pharmacists and Licensed by the Regulatory Agency, Pharmaceutical Council of Nigeria (PCN); (*b*) registered PPMVs who are not pharmacists but are registered by PCN to sell over-the-counter medicines only, and (*c*) Unregistered PPMVs. The registered medicine retailers have been used as platforms to expand access to care given their ubiquitous nature with capacity building programmes provided for them by several organizations on different diseases. They have also been trained to report adverse drug events through their participation in the National Pharmacovigilance programme and antimalarial subsidy programme such as the affordable medicines facility for malaria (AMFm).^9^

To ensure effective case management of malaria with ACTs in all settings, it is imperative that parasitological confirmation of malaria be introduced at the point where malaria medicines are sold or dispensed. This is major challenge in African and Asian countries. The feasibility of introducing malaria rapid diagnostic tests (RDTs) to medicine retailers in the informal private sector have been severally described in some African and Asian countries.^10-15^ The outcome of these showed that malaria RDTs are easy to perform by medicine retailers with great potentials of expanding access to diagnosis in line with the diagnosis and treatment policy requirement. Studies on malaria parasitaemia among clients that patronized medicine retailers in Tanzania showed that a considerable number of these clients would not need antimalarial medicines.^16,17^ In Nigeria, current regulatory policy on the PPMVs does not permit them to perform minimally invasive procedures. This includes the performance of malaria RDT that requires fingerpicking to collect blood. Data on ACT misuse emanating from the prevalence of malaria among persons suspected to have malaria who purchased antimalarials from the PPMVs is needed to provide evidence for a policy change that will institute parasitological confirmation of malaria at settings where antimalarial medicines are sold. This study reports ACT misuse among adult clients suspected to have malaria that purchased malaria medicines from PPMVs in Nigeria.

## Methods

### Study Sites and Population

**Figure 1:**
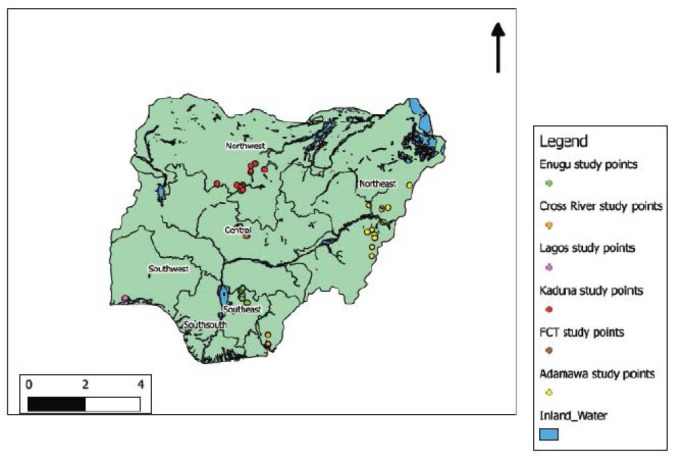
Study Sites in Five Geopolitical Zones Where Clients That Purchased ACTs Were Tested in Nigeria. Abbreviation: ACts, artemisinin combination therapies.

This study was conducted in retail outlets of PPMVs in five states spread across five geopolitical zones of the country including the Federal Capital Territory, Abuja. These are: Adamawa (North-East zone); Cross River (South-South), Enugu (South-East), Lagos (South-West), Kaduna (North-West) and the Federal Capital Territory of Abuja. Nigeria, with an estimated population of 180 million people, consists of six geopolitical zones with 36 states and the Federal Capital Territory (FCT), Abuja. The study sites were unevenly distributed in five geopolitical zones namely: North-Central, North-West, South-South, South-East and South West; and are spread across various ecological zones (Figure 1). The population studied were made of adult male and females, 20 years and above who gave consent to participate. The study was conducted between November and December 2012 during the dry season when transmission of malaria is low.

### Study Design

A cross-sectional, multi-stage purposive sampling was used to select the states and the PPMVs that participated in the study while all eligible clients that qualified for the study were randomly selected based on odd number presentation. Essentially, a client was skipped after a previous enrollment until the third eligible client arrived so as to remove bias in the client selection. This study was conducted within a larger study on the feasibility of conducting malaria diagnosis in private sector retail facilities.

The selection of the PPMVs was done in three stages: Stage 1: This involved the selection of one state from each of the six geopolitical zones. These states represented different geographic and linguistic-ethnic regions of the country, malaria zones within the country and preponderance of PPMVs. Stage 2: Rural and urban local government areas (LGAs) were selected in each of the states. Stage 3: The PPMVs were selected from a list of medicine stores partnering with the Global Fund Malaria Project implemented in Nigeria by The Society for Family Health. The criteria for selecting PPMVs study included availability in the area most of the time, acceptability by the community, educational level (minimum of secondary school education), and consent to participate.

About 20 registered PPMVs were selected from each of the selected states through a computer-generated randomization of registered PPMVs from which numbers representing facility names were selected. These outlets were grouped into clusters of 6 per state. All the outlets used for the study were mapped using the global positioning system (GPS) [Garmin Corporation]. Laboratory personnel were trained on how to prepare thick and thin blood smears for malaria microscopy. Research Assistants who conducted exit interviews with clients who purchased an ACT at each study point were also trained. The research assistants were health workers with clinical training and have experience in managing patients at the lower level of care.

The minimum sample size required for the exit interview component of the study was calculated by anticipating a 15% level of change, assuming that the current level of quality provided is 25%. With a design effect fixed at 1.5, level of significance at 5%, power of test at 80% and adjusting for a 10% non-response, a minimum sample of 179 per state was achieved. This was rounded up to a total of 200 for each state representing a geopolitical zone. Therefore, in the six selected states it was estimated that a total of 1200 clients who purchased ACTs from the various PPMV outlets would be interviewed and malaria parasite test performed.

### Sample and Data Collection

The PPMVs were grouped in clusters with an interviewer and laboratory scientist assigned to each group. PPMVs alerted the laboratory scientist/research assistants who were within the vicinity when a client visited the outlet seeking malaria treatment. A minimum of 10 clients, who visited each of the PPMVs, purchased ACT for the treatment for malaria and who gave consent was enrolled for the study. The inclusion criteria used in enrolling the clients that participated were: adults, 20 years and above; patients requiring malaria treatment (ACTs) and written consent. The exclusion criteria were: non-adults, less than 20 years of age; refusal to consent; clients not buying medicine for use; and clients with severe illness. The exit interview was done using a questionnaire that captured information on the age, gender, educational level, economic status, presenting symptoms, etc.

### Malaria Microscopy

Thin and thick blood films were prepared on the same slide by trained laboratory scientists. The slides were made in duplicates for each client and labeled appropriately. Thin film was fixed by dipping the thin film end of the slide in a beaker containing absolute methanol for 1-2 seconds and air-dried. All prepared slides were packaged in a slide box and immediately sent to the ANDI Centre of Excellence in Malaria Diagnosis, College of Medicine of the University of Lagos, Lagos, Nigeria, where they were stained with 3% Giemsa stain at pH 7.2. Two WHO-certified microscopists read the Giemsa-stained slides. Discordant readings in parasite detection and parasite densities were resolved before the final result was taken. A total of 200 oil immersion fields (OIFs) were read before a slide was declared negative. The mean parasite densities obtained by two microscopists was used to determine the parasite density per client provided the percentage discrepancy of the two readings was less than 20%. The absolute parasite density (parasite per microliter of blood) was calculated by multiplying the number of parasites counted with an estimated 8000 leucocytes and divided by the relative leucocytes counted. In all cases, a third WHO-certified microscopist served as the tiebreaker.

Feedback on the outcome of the malaria microscopy test was shared with the PPMVs and the clients that were tested.

### Data Analysis

The data obtained from the study was entered and verified using the data management software, CSPro 2.6 and subsequently imported into SPSS (version 18) for statistical analysis. Test for association was done using the Pearson chi-square test at .05 significant levels.

## Results

### Characteristics of Registered Drug Shop Owners (PPMVs)

A total of 119 PPMVs from the 130 that were trained participated in the study from the six states selected from the six geopolitical zones of the country. These included 21 PPMVs from Lagos state, Enugu state (19), Cross River (20), FCT (16), Adamawa state (25), and Kaduna state (18).

### Characteristics of the Clients

The 1279 respondents, 20 years and above, who sought treatment for their illness from the PPMV outlets were: 188 from Enugu State, Cross River (229), FCT (213), Kaduna (256), Adamawa (188), and Lagos State (205). The median age of respondents was 32 years. The proportion of males in the population was 55% while females were 45%. Sixty-two percent of the respondents were married. The educational level of respondents who participated in the survey showed that 43% had attained a secondary level of education, one in four (25%) of the respondents had attained a higher level of education (Table 1).

**Table 1:** Characteristics of Clients that Purchased Antimalarial Medicines From the PPMVs

**Description, n = 1279**	**No. (%)**
State	
Adamawa	186 (14.8)
Cross River	228 (18.2)
Enugu	180 (14.3)
FCT	210 (16.7)
Kaduna	248 (19.7)
Lagos	204 (16.2)
Gender	
Male	685 (54.5)
Female	570 (45.4)
Marital status	
Married	769 (62.2)
Single	412 (33.3)
Divorced	43 (3.5)
Age	
20–24 years	178 (13.9)
25–29 years	216 (16.9)
30–34 years	234 (18.2)
35–39 years	134 (10.5)
40–44 years	125 (9.80)
45–49 years	185 (14.5)
50 years and above	207 (16.2)
Education	
None	149 (11.6)
Quranic only	51 (4.0)
Primary	225 (17.6)
Secondary	546 (42.7)
Higher	308 (24.1)

Abbreviations‏: PPMVs, patent and propriety medicine vendors; FCT, Federal Capital Territory.

### Symptoms Presented by the Tested Clients at the Shops of PPMVs

**Figure 2:**
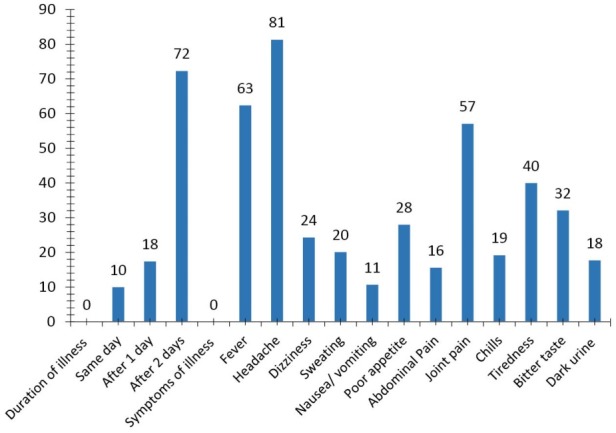
Symptoms of Clients that Purchased ACTs From PPMVs. Abbreviations: PPMVs, patent and propriety medicine vendors; ACTs, artemisinin combination therapies.

The visit to the PPMVs in most cases was after 48 hours of initial symptoms (72%) (Figure 2). The reported symptoms by most of the respondents can be associated with, but may not be specific to malaria. Fever (63%), headache (81%), joint pains (57%), tiredness (40%), bitter taste (32%), and poor appetite (28%) were the most common symptoms reported by clients that sought for treatment from the PPMVs (Figure 2). Most of the clients sought treatment after two days of experiencing symptoms.

### Prevalence of Malaria Among Clients that Visited and Purchased Malaria Medicines From PPMVs

In general, blood smear results by microscopy across the study sites showed that of the 1279 clients tested, 107 (8.4%) were confirmed to have malaria. The confirmed malaria rates in the states were: Enugu, 20 (10.6%), Cross River 8 (3.5%), FCT 16 (7.5%), Kaduna 15 (6%), Adamawa 30 (16%), and Lagos 18 (8.8%). The distribution of *Plasmodium* species in this study was: *Plasmodium falciparium* (99%), *P. malariae* (0.1%) while the remaining were mixed infections with *Pf* (*P. malariae* and *P. ovale*). Further more, majority of the parasite stages encountered were trophozoites (91%) and other stages were mix of both trophozoite and gametocytes.

### Malaria Symptoms by Clients at Presentation at Medicine Retail Shops

In this study, majority of the clients visited PPMVs after two days of having malaria-like symptoms. Those that purchased antimalarial medicines at the first day of having symptoms were lower compared to those that visited the PPMVs and purchased medicines a day after symptoms were observed (Figure 2). The most common symptoms of the client at presentation at PPMVs were: Headache (81%), fever (63%), joint pains (57%), and tiredness (40%) while the least was nausea (11%) (Figure 2).

### Symptoms of Clients With Confirmed Malaria by Microscopy

None of the most common symptoms at presentation by the clients had significant correlation with malaria by microscopy. Headache, fever, joint pains and tiredness were not significantly associated with malaria among clients, 20 years and above that were studied. However, nausea/vomiting, poor appetite, chills, bitter taste in the mouth and dark urine were significantly associated with malaria among the clients (*P* < .05) but not fever (*P* = .056) (Table 2). Of the 796 clients that had fever/symptoms of fever, 76 (9.5%) had malaria parasites while 720 (90.5%) were aparasitaemic (Table 2).

**Table 2:** Symptoms of Clients Who Purchased ACTs in Retail Medicine Shops and Malaria

**Symptoms**	**Microscopy Positive** **No. (%)**	**Microscopy Negative** **No. (%)**	***P*** ** Value**
	**(n = 107)**	**(n = 1167)**	
Fever	76 (71.0)	720 (61.7)	.056
Headache	87 (81.3)	950 (81.4)	.980
Dizziness	25 (23.4)	286 (24.5)	.792
Sweating	25 (23.4)	232 (19.9)	.390
Nausea/vomiting	19 (17.8)	119 (10.2)	.016*
Poor appetite	44 (41.1)	314 (26.9)	.002*
Abdominal pain	20 (18.7)	180 (15.4)	.374
Diarrhoea	6 (5.6)	38 (3.3)	.202
Shortness of breath	6 (5.6)	59 (5.1)	.804
Congestion	4 (3.7)	31 (2.7)	.512
Dry cough	12 (11.2)	166 (14.2)	.390
Convulsion	2 (1.9)	9 (0.8)	.240
Joint pain	67 (62.6)	662 (56.7)	.239
Chills	35 (32.7)	211 (18.1)	.000*
Yellow eyes	9 (8.4)	71 (6.1)	.342
Tiredness	45 (42.1)	474 (40.6)	.772
Bitter taste in the mouth	45 (42.1)	365 (31.3)	.022*
Dark urine	28 (26.2)	199 (17.1)	.018*

Abbreviation: ACTs, artemisinin combination therapies.

## Discussion

One of the control strategies for malaria is effective case management that requires early diagnosis and prompt treatment with ACTs. Current malaria case management recommendation requires prior parasitological confirmation of all suspected cases of malaria before treatment by either microscopy or malaria RDTs. The need for prior testing of suspected malaria cases is critical given the poor specificity of clinical criteria in the diagnosis of malaria in light of several aetiologies of fever. The implementation of this recommendation in medicine retailer’s outlets is just emerging and the evidence on malaria confirmed rates in the informal private health sector that is controlled by the PPMVs is scarce. The changing epidemiology of malaria from high endemicity to hypoendemicity implies that widespread presumptive treatment may lead to over-use of antimalarial medicines given reduced malaria rates. This study provides evidence for policy to curb the irrational use of ACTs among clients who seek treatment from PPMVs without prior parasitological confirmation of malaria before treatment or sales of antimalarial medicines.

Malaria medicines are directly requested by clients from PPMVs who believed they had malaria and in most cases, the PPMVs would recommend antimalarial medicines following complaints from their clients. The request for ACTs by clients or prescription by PPMVs was done based on the widespread belief that fever, in addition to other symptoms, such as headache and tiredness, for example are indicators of malaria. The confirmation of malaria requires the detection of *Plasmodium* by microscopy or the appropriate *Plasmodium* antigen by malaria RDTs in patients with presenting symptoms. Thus, the non-establishment of *Plasmodium* parasitaemia or antigenemia by malaria RDTs with concomitant use of ACTs is tantamount to ACT misuse. The 8.4% prevalence of Malaria among adult clients confirmed by microscopy provides the first evidence in Nigeria and showed a high level of misuse of ACTs. We have shown in this study that fever was not significantly associated with malaria; yet it was an indicator to treat for malaria. It was one of the symptoms that accounted for 90.5% misuse of ACTs among the adult clients in our study population.

The consequences of presumptive use of ACTs among the studied group could delay early intervention in non-malarial febrile illness, as patients may not receive the appropriate treatment for their condition. Similar observations have been reported in some African countries. The prevalence of malaria parasitaemia among clients seeking treatment for fever or malaria at drug stores in rural Tanzania showed that 63.8% of them were clinically diagnosed for malaria while malaria blood film confirmation was 24.2%.^15^ Another report that underscored the need for prior testing before sales of antimalarial medicines from two regions of Tanzania among 777 clients from 73 drug shops showed that parasitologically-confirmed malaria was 12%.^16^

Furthermore, a systematic review of reports from 16 African countries showed a considerable reduction in the proportion of fevers associated with *Plasmodium falciparum* parasitaemia and this justified the policy change from presumptive antimalarial treatment of children with fever to laboratory diagnosis before treatment.^17^

There is an urgent need to expand access to parasitological confirmation of malaria^18^ in the informal private retail sector in countries where they provide service to large number of people. Already, a number of published studies have demonstrated feasibility of using malaria RDTs in drug shops.^9-14^ Ikwuobe et al^19^ highlighted possible over-treatment of malaria with a reported malaria prevalence of 13.6% in Gwagwalada, Nigeria, using malaria RDTs among patients that visited community pharmacies. Substantial symptoms’ overlap has also been reported between malaria and other common illnesses caused by viral and bacterial infections^20^ while drastic reduction in antimalarial drug consumption are positive outcome of malaria confirmation before treatment as reported in Tanzania, Zambia, and Senegal.^21-23^

## Conclusion

The level of ACT misuse among adult clients suspected to have malaria that visited selected PPMV shops in Nigeria was high. Some common symptoms that are widely used as an indicator of malaria such as fever, headache, and tiredness were not significantly associated with confirmed malaria parasitaemia among the adult clients that purchased antimalarial medicines. Early detection and prompt treatment is critical for effective malaria case management. Consequently, to prevent the eventual fatality of patients that may present with other life-threatening non-malarial febrile conditions that could be erroneously managed as malaria, promotion of prompt and effective management of malaria, through parasitological confirmation of suspected malaria at the points where malaria medicines are sold or dispensed is imperative. A strategic framework for parasitological confirmation of suspected malaria cases by PPMVs should be developed, implemented and monitored. Access to malaria diagnosis will substantially reduce excessive and unnecessary consumption of ACTs.

## Acknowledgements

This study was funded by the Global Fund to Fight HIV & AIDS, Tuberculosis, and Malaria (GFATM). We thank the clients that consented to participate in this study after the purchase of antimalarial medicines, the patent and propriety medicine vendors (PPMVs) whose shops were used to implement the study, Medical Laboratory Scientists and Nurses that interfaced with the clients and the staff of The ANDI Centre of Excellence for Malaria Diagnosis/International Microscopy & RDT Quality Assurance Centre, College of Medicine of the University of Lagos, Lagos, Nigeria for performing the malaria microscopy.

## Ethical issues

The study was conducted in accordance with best practices encapsulated in the Helsinki Declaration of the World Medical Association of 1964 as amended in 2008. All participants involved in the study gave informed consent and they were free to withdraw at any point of the study. This study was approved by the National Health Research and Ethics Committee (NHREC/01/01/2007-30/10/2012b).

## Competing interests

Authors declare that they have no competing interests.

## Authors’ contributions

All authors designed the study, participated in data collection, analyses and write up of manuscript. Every author made inputs to the revision of the manuscript.

## Authors’ affiliations

^1^Society for Family Health, Abuja, Nigeria. ^2^ANDI Centre of Excellence for Malaria Diagnosis, College of Medicine, University of Lagos, Lagos, Nigeria.

## Key messages

**Implications for policy makers**

- Policy permitting patent and propriety medicine vendors (PPMVs) to perform parasitological testing with rapid diagnostic tests (RDTs) before sales of antimalarial medicines will ensure an all-inclusive public and private health sectors’ implementation of the testing, treatment and tracking policy.
- Parasitological confirmation of malaria by PPMVs who are ubiquitous and attend to a majority of persons with fever will reduce the misuse of antimalarial medicines and promote appropriate management of non-malarial fevers.
- Malaria testing with RDTs by PPMVs through collaborative partnerships will promote effective malaria case management and expand access to universal malaria testing.
- The development of a framework that fully integrates PPMVs in malaria case management through regulation and registration, capacity building, monitoring and supervision, will expand access to effective malaria case management.

**Implications for the public**

The symptoms of malaria are non-specific as it is similar to symptoms of a number of infections and other illness that are life threatening. Therefore, it is imperative for clients with malaria-like symptoms to first confirm if they had malaria or not so that their condition could be properly managed and not assume that artemisinin combination therapies (ACTs) would be useful when other non-malarial medicines could have been dispensed. This assumption could be fatal due to delay in receiving appropriate treatment. Thus, the performance of quality assured rapid diagnostic tests (RDTs) by patent and propriety medicine vendors (PPMVs) will reduce mortality, morbidity of other non-malarial febrile illnesses, and on the long run, the cost of malaria management. Consequently, it is critical that persons or clients with suspected symptoms of malaria be tested or should demand for prior testing before the purchase and use of ACTs.
